# Sex-Specific Cytokine Responses and Metabolic Adaptation to Weight Loss in Obesity with Insulin Resistance

**DOI:** 10.3390/nu18121982

**Published:** 2026-06-18

**Authors:** Maria Dydoń, Anna Birková, Paweł Dolibog, Beáta Čižmárová, Beáta Hubková, Zenon Czuba, Paulina Zalejska-Fiolka, Agata Dydoń, Sławomir Kasperczyk, Bronisława Skrzep-Poloczek, Jolanta Zalejska-Fiolka

**Affiliations:** 1 Department of Biochemistry, Faculty of Medical Science in Zabrze, Medical University of Silesia, 40-055 Katowice, Poland; m.dydon@nzozkruszyna.pl (M.D.); s91883@365.sum.edu.pl (P.Z.-F.); a.dydon@nzozkruszyna.pl (A.D.); skasperczyk@sum.edu.pl (S.K.); jzalejskafiolka@sum.edu.pl (J.Z.-F.); 2 Department of Medical and Clinical Biochemistry, Pavol Jozef Šafárik University, 040 11 Košice, Slovakia; beata.cizmarova@upjs.sk (B.Č.); beata.hubkova@upjs.sk (B.H.); 3 Department of Biophysics, Faculty of Medical Sciences, Medical University of Silesia, 41-808 Zabrze, Poland; pawel.dolibog@sum.edu.pl; 4 Department of Microbiology and Immunology, Faculty of Medical Science in Zabrze, Medical University of Silesia, 40-055 Katowice, Poland; zczuba@sum.edu.pl; 5 Scientific Research Facility, Branch in Bielsko-Biała, Medical University of Silesia, 40-055 Katowice, Poland; bskrzep-poloczek@sum.edu.pl

**Keywords:** cytokines, insulin resistance, sex differences, weight-reduction program, visceral fat

## Abstract

**Background/Objectives**: Obesity-related insulin resistance is accompanied by chronic low-grade inflammation, but the extent to which weight loss modifies circulating cytokines in a sex-specific manner remains insufficiently understood. The aim of this study was to assess sex-specific cytokine responses and metabolic adaptation in adults with obesity and insulin resistance following a six-month weight-reduction program (WRP). **Methods**: Thirty-six participants (24 women and 12 men) with a value of Homeostatic Model Assessment of Insulin Resistance (HOMA-IR) ≥ 2 underwent an individualized low-calorie diet combined with moderate physical activity and health education. Anthropometric, body composition, biochemical, oxidative stress, and cytokine parameters were evaluated before and after the intervention. **Results**: Both women and men showed significant reductions in body mass, Body Mass Index (BMI), waist circumference, visceral fat area (VFA), body fat mass (BFM), fasting glucose, HOMA-IR, modified Atherogenic Index of Plasma (new-AIP), malondialdehyde (MDA), and Oxidative Stress Index (OSI). Women additionally showed significant decreases in fat-free mass (FFM), skeletal-muscle mass (SMM), total body water (TBW), glycated hemoglobin A_1c_ (HbA1c), and triacylglycerols, whereas cholesterol in high-density lipoproteins (HDL-C) increased significantly in men. Cytokine changes were selective rather than uniform. Interleukin-1 receptor antagonist (IL-1ra), Interleukin 6 (IL-6), and Tumor Necrosis Factor alpha (TNF-α) decreased in both women and men. In sex-stratified analyses, IL-1β decreased significantly only in women, whereas IL-7 decreased significantly only in men. ClinicalTrials.gov Registration: [NCT07645105] (retrospectively registered on [11 June 2026]). **Conclusions**: A 6-month lifestyle-based weight-reduction program in adults with overweight or obesity and insulin resistance was associated with metabolic improvement, reduced oxidative stress, and partial attenuation of obesity-related low-grade inflammation. The observed cytokine and metabolic changes suggest sex-related patterns of immunometabolic adaptation to weight reduction. However, these findings should be interpreted cautiously because of the relatively small sex-stratified subgroups and the number of cytokine endpoints analyzed, and they require confirmation in larger, sex-balanced studies.

## 1. Introduction

Obesity is a major global health challenge. In 2022, 43% of adults worldwide were overweight, and 16% were living with obesity. Projections indicate a further increase in the coming decades [1,2]. Beyond excess energy storage, obesity is now regarded as a systemic metabolic, endocrine, and immune disorder. In obesity, adipocyte hypertrophy, tissue hypoxia, extracellular matrix remodeling, and immune cell recruitment promote persistent, low-grade inflammation—or metaflammation [3,4,5,6,7,8,9]—which contributes to insulin resistance, dyslipidemia, type II diabetes, cardiovascular disease, and other obesity-related complications [4,5,6,7,8,9].

Obesity-related metaflammation is reflected by dysregulated cytokine and chemokine signaling. Mediators, such as IL-6 and TNF-α, participate in insulin resistance, whereas chemokines, such as Monocyte Chemoattractant Protein-1 (MCP-1), support monocyte/macrophage recruitment into adipose tissue and sustain inflammatory loops [4,5,6,7,8,9]. At the same time, obesity is associated with a broader remodeling of innate and adaptive immunity, including an altered T helper cell (Th) Th1/Th2/Th17 balance, impaired regulatory pathways, and changes in growth-factor signaling. Multiplex cytokine assessment may, therefore, provide a broader view of immunometabolic activation than single-marker analyses. IL-17-related pathways are also of interest because they may connect adipose-tissue inflammation with disturbed glucose homeostasis and metabolic regulation [10,11].

BMI alone does not sufficiently describe obesity-related metabolic risk. Visceral adiposity is particularly relevant because visceral fat is more metabolically active and more closely linked to inflammation, insulin resistance, and atherogenic dyslipidemia than overall adiposity. Therefore, interpretation of inflammatory responses to weight reduction should include not only the amount of weight lost, but also the biological quality of weight loss, reflected by body-composition parameters such as VFA, percentage of body fat (PBF), FFM, SMM, and body-water compartments.

The importance of sex-specific cytokine responses is particularly relevant in obesity with insulin resistance, because biological sex may influence both the source and the metabolic consequences of low-grade inflammation. Differences in visceral and subcutaneous fat distribution, adipose-tissue expandability, sex-hormone signaling, and skeletal-muscle metabolism may modify cytokine production and the relationship between inflammatory mediators and glucose–insulin homeostasis [12,13]. Therefore, the same degree of weight reduction may not necessarily translate into the same inflammatory and metabolic responses in women and men. In this context, cytokine profiling may help identify whether lifestyle-induced weight loss attenuates systemic inflammation through common mechanisms or through sex-related immunometabolic patterns. This issue remains clinically relevant because obesity treatment is still usually monitored mainly by body weight, BMI, and routine metabolic markers, whereas inflammatory adaptation and the biological quality of weight loss are rarely assessed in a sex-stratified manner.

Lifestyle-based weight reduction can attenuate obesity-related metabolic and inflammatory disturbances; however, the published evidence remains heterogeneous and depends on intervention type, duration, study population, and the range of inflammatory markers assessed [14,15,16]. Thus, the systemic inflammatory response to moderate weight loss remains incompletely characterized, particularly when broad cytokine panels are interpreted together with insulin resistance and detailed body-composition data.

In our previous studies using the same weight-reduction model, we showed that the biological quality of weight loss depends not only on the magnitude of body-mass reduction but also on glycemic status and tissue-specific changes. Impaired glycemic control was associated with less favorable reductions in visceral adiposity, greater losses of skeletal-muscle mass and body water, persistent atherogenic risk, and distinct oxidative adaptations in serum and erythrocytes during weight reduction [17,18,19,20]. These findings suggest that inflammatory mediators may contribute to the heterogeneity of metabolic adaptation to lifestyle intervention.

To our knowledge, relatively few studies have evaluated lifestyle-induced cytokine changes in insulin-resistant obesity using a sex-stratified approach combined with detailed body-composition, glycemic, lipid, and oxidative-stress profiling. Therefore, the present study aimed to assess sex-stratified cytokine responses in adults with obesity and insulin resistance participating in a 6-month weight-reduction program. A broad multiplex cytokine panel was analyzed together with anthropometric, body-composition, lipid, glucose–insulin, and oxidative-stress parameters. We hypothesized that moderate weight loss would be associated with selective, rather than uniform, changes in circulating inflammatory mediators; that these changes would relate more closely to visceral adiposity and insulin resistance than to BMI alone; and that the pattern of these associations would show sex-related patterns in women and men.

## 2. Materials and Methods

The study included 36 individuals with obesity (people with obesity, PWO) (24 females and 12 males) with insulin resistance (HOMA-IR ≥ 2) who voluntarily participated in a 6-month WRP. The study involved the collection of data at two times: before the weight-reduction program (B-WRP) and post-WRP (P-WRP). The program consisted of a balanced, individualized, low-calorie diet; moderate physical activity; and health education tailored to the participants’ needs. PWO were under the care of the Metabolic Clinic in Miasteczko Śląskie, Poland.

The HOMA-IR was calculated as: (fasting insulin [µIU/mL] × fasting glucose [mg/dL])/405 [21]. The study was carried out in line with the Declaration of Helsinki and received approval from the Medical University of Silesia Ethics Committee in Katowice, Poland, No. KNW/0022/KB1/19/I/16. Written informed consent was obtained from all participants for study participation as well as for the publication of the findings. To reduce the risk of bias, all samples were anonymized and assigned numerical codes before laboratory testing and statistical evaluation. At the time the study was initiated in 2016, prospective registration in a public clinical trial registry was not considered necessary under the applicable national regulations or the requirements of the Institutional Bioethics Committee. The study was designed and conducted as a prospective observational cohort investigation within routine metabolic outpatient practice and did not involve any investigational medicinal products, medical devices, dietary supplements, surgical procedures, or other experimental interventions. The study was retrospectively registered at ClinicalTrials.gov on 11 June 2026 (Identifier: NCT07645105). Retrospective registration was undertaken to align the study with current international standards for research reporting and publication and to ensure public accessibility of the study information. The registration provides a publicly available record of the study design, objectives, methodology, and outcome measures, thereby supporting openness and accountability in the reporting of the study findings.

The sample size calculation was based on the primary endpoint of the study, namely weight reduction. The previous literature [22] and our earlier findings [17,18,19,20] indicate that a 5–15% reduction in body weight over approximately six months is associated with improvements in cardiometabolic risk factors. On this basis, it was estimated that a minimum of 11 participants per group would be required to achieve statistical significance at *p* = 0.05 with 80% statistical power. Furthermore, the calculation accounted for an anticipated dropout rate of 20%.

### 2.1. Structured Weight-Reduction Intervention

Participants entered a six-month comprehensive weight-reduction program initiated in response to evidence indicating that weight loss contributes to improvements in lipid profiles, cardiometabolic risk factors, hyperglycemia, and type II diabetes. At baseline, all individuals reported a diet of suboptimal quality, characterized by high intake of saturated fatty acids, salt, simple sugars, refined grains, and processed foods, along with low consumption of antioxidant-rich fruits and vegetables and insufficient physical activity. During the initial visit, each participant underwent a medical evaluation and completed a detailed questionnaire covering obesity history, dietary habits, food preferences, comorbidities, and habitual physical activity. Individual metabolic parameters were calculated, including basal metabolic rate (BMR), total daily energy expenditure (calculated by the comprehensive metabolic panel, CPM), and the recommended daily energy deficit (DDE). A moderate caloric restriction corresponding to approximately 15% of total daily energy expenditure was prescribed.

Dietary recommendations were developed in accordance with the guidelines of the Polish National Food and Nutrition Institute (IŻŻ) and the World Health Organization (WHO). Participants received individualized nutritional plans designed to ensure balanced macronutrient distribution: 25–35% of total energy intake from fats (with saturated fatty acids limited to <10% and 3–6% from mono- and polyunsaturated fatty acids, primarily from vegetable and fish oils), 45–65% from carbohydrates (with free sugars restricted to <10%), and 10–15% from protein sources of both animal and plant origin. Particular emphasis was placed on increasing daily vegetable intake to approximately 500 g and fruit intake to 200 g. Participants were encouraged to consume five properly balanced meals per day. When necessary, low- or medium-glycemic index snacks, such as fruits or vegetables, were recommended. Participants also received recommendations to maintain optimal daily water intake.

In addition to dietary modification, participants were instructed to perform moderate-intensity physical activity at least three times per week, with each session lasting approximately 40 min.

Follow-up visits were scheduled every 3–4 weeks. During these visits, body weight and waist and hip circumferences were measured, and adherence to dietary composition and caloric targets was regularly assessed and recorded. Participants were encouraged to keep self-reported dietary and physical-activity diaries throughout the intervention. These diaries included information on meal patterns, food choices, caloric intake when available, deviations from the prescribed diet, physical activity, and difficulties encountered during the preceding month. At each follow-up visit, the diaries and the participants’ reported adherence were reviewed, and individualized feedback was provided. Ongoing motivational support included education on calorie calculation, food quality assessment, correction of dietary mistakes, and sustainable lifestyle practices. Physical activity was monitored on the basis of participants’ self-reports and diary entries, but it was not objectively quantified using accelerometers, pedometers, or other wearable devices.

Progress was monitored throughout the intervention period until participants achieved either a healthy body weight or a 5–15% reduction from baseline body mass, with the overall intervention lasting approximately six months.

### 2.2. Participant Selection and Study Groups

This was a single-center, prospective before-and-after intervention study conducted at the Metabolic Clinic in Miasteczko Śląskie, Poland. The study was designed to assess within-subject changes after a structured 6-month WRP; therefore, each participant’s baseline values obtained before the intervention served as the reference point for post-intervention assessment. This approach was chosen because biochemical, inflammatory, and body-composition parameters exhibit substantial inter-individual variability among people with overweight/obesity and insulin resistance. Within-subject comparison, therefore, allowed for the evaluation of individual metabolic adaptation to weight reduction. However, the absence of a separate non-intervention or weight-stable control group limits causal interpretation and is acknowledged as a limitation of the study. The present analysis was derived from approximately 300 patients diagnosed as overweight or obese on the basis of medical examination and body-composition assessment, including BMI, waist-to-hip ratio (WHR), body fat mass, and the index of central obesity (ICO). All patients were characterized by at least one parameter indicating excessive body mass or visceral obesity. None of the patients included in the final analysis were taking medication known to affect lipid or glucose metabolism. The final analytical cohort consisted of 36 adults with overweight/obesity and insulin resistance, including 24 women and 12 men. Participants were eligible for the present analysis if they fulfilled the predefined inclusion criteria, completed the 6-month WRP, and had complete anthropometric, body-composition, biochemical, oxidative-stress, adipokine, and cytokine data available at both time points. The reduction from the initially evaluated clinical population to the final analytical cohort resulted mainly from strict inclusion and exclusion criteria, discontinuation of follow-up visits, non-adherence to dietary and physical-activity recommendations, later identification of treatments affecting glucose and/or lipid metabolism, and incomplete laboratory or cytokine datasets.

The inclusion criteria were: BMI > 25 kg/m^2^; insulin resistance defined as HOMA-IR ≥ 2; no pharmacological treatment known to affect glucose or lipid metabolism; completion of the 6-month WRP; complete data at both time points; and written informed consent. A HOMA-IR threshold of 2 was used, where HOMA-IR < 2 indicates no insulin resistance and HOMA-IR ≥ 2 indicates insulin resistance [23].

The exclusion criteria were: lack of consent to participate in the study; severe hepatic, renal, respiratory, or circulatory insufficiency; disturbances of consciousness; treatment-resistant depression; chronic alcohol abuse; pregnancy; history of serious nervous system injury; implanted cardiac pacemaker; pharmacological treatment affecting glucose and/or lipid metabolism; incomplete follow-up; or incomplete laboratory/cytokine data.

Because the aim of this study was to assess sex-stratified metabolic and inflammatory adaptation to weight reduction, participants were categorized by biological sex into female (*n* = 24) and male (*n* = 12) subgroups. The smaller male subgroup reflects the composition of the final eligible cohort after applying the strict criteria, requiring completion of follow-up, and including only participants with complete cytokine datasets. This relatively small and sex-imbalanced sample is acknowledged as an important limitation of the study.

### 2.3. Biomarker Assessments

Fasting blood samples were collected from the cubital vein using clot activator tubes (2.7 mL) to obtain serum. Following centrifugation for 10 min at 3000 rpm at 4 °C, the serum was aliquoted and preserved at −80 °C within the Department of Biochemistry, Faculty of Medical Science, Medical University of Silesia, Poland. Throughout the study, the analytical procedures adhered to continuous intra-laboratory quality control measures.

HbA1c and serum glucose levels were measured using a Miura 200 DA biochemical analyzer (I.S.E. S.r.l., Guidonia Montecelio, Italy). Glucose was assessed via the glucose oxidase method. HbA1c was analyzed in EDTA whole-blood samples using the latex-enhanced immunoturbidimetric technique (Human GmbH, Wiesbaden, Germany). Additionally, serum insulin levels were measured with INS-IRMA kits (KIP1251-KIP1254, DIA Source Immuno Assays S.A., Louvain, Belgium). The repeatability, indicated by the within-run coefficient of variation, was 0.6% for glucose, 1.4% for HbA1c, and 4.1% for insulin. The between-run coefficient of variation for these parameters was 1.6%, 3.1%, and 7.7%, respectively.

Serum parameters of the lipid profile (total cholesterol, cholesterol in low-density lipoproteins (LDL-C), HDL-C, and triacylglycerols (TG)) were determined using a biochemical analyzer Miura 200 DA (I.S.E. S.r.l., Italy). Repeatability (within-run precision coefficients of variation) for total cholesterol, TG, HDL-C, and LDL-C were 0.9%, 0.8%, 2.4%, and 2.2%, respectively. Reproducibility (between-run precision coefficients of variation) for the above-mentioned parameters were 1.7%, 2.1%, 3.8%, and 3.5%, respectively. The analysis of lipoprotein fractions and sub-fractions in fasting serum with cholesterol levels exceeding 2.59 mmol/L (100 mg/dL) was performed using the Lipoprint Lipoprotein Subfractions Testing System (Quantimetrix, Redondo Beach, CA, USA), as previously described [23].

New-AIP was calculated as [23]:

$$\mathrm{AIP}\;\left(\mathrm{new}\;\mathrm{formula};\;\mathrm{new}\text{-}\mathrm{AIP}\right)\;=\;\log\frac{\mathrm{TG}}{\mathrm{anti}\text{-}\mathrm{atherogenic}\;\mathrm{HDL}\text{-}C}$$

where TG is the triacylglycerol concentration, and anti-atherogenic HDL-C is the anti-atherogenic high-density lipoprotein cholesterol (subfractions 1–3).

Furthermore, we evaluated the OSI, which is the ratio of total oxidant status (TOS) to total antioxidant capacity (TAC). TOS was measured using Erel’s automated method (2005) [24]. This method involves the oxidation of ferrous ions (Fe^2+^) to ferric ions (Fe^3+^) in an acidic environment in the presence of oxidants. Ferric ions then react with xylenol orange to produce a colored complex detectable at 560 nm. Reagent I included 150 µM xylenol orange, 140 µM NaCl, and 1.35 M glycerol in 25 mM H_2_SO_4_. Reagent II consisted of 5 mM ferrous ammonium sulfate and 10 mM o-dianisidine dihydrochloride. Hydrogen peroxide was used for calibration, and results were expressed as µmol H_2_O_2_ equivalents per gram of protein. Replicate measurements confirmed analytical precision, with an inter-assay coefficient of variation (CV) below 7%. TAC was determined using the automated colorimetric method by Erel (2004) [25], which assesses the antioxidant ability to neutralize the ABTS^+^ radical cation (2,2′-azinobis(3-ethylbenzothiazoline-6-sulfonate)), thereby decreasing the blue–green color intensity measured at 660 nm. The assay reagents included 10 mM ABTS in acetate buffer (pH 5.8) and 2 µM hydrogen peroxide to generate radicals. Trolox (6-hydroxy-2,5,7,8-tetramethylchroman-2-carboxylic acid) served as the calibration standard, with results expressed as Trolox equivalents per gram of protein. All tests were performed in duplicate, with intra-assay CVs below 5%.

Adipokine levels were quantified in serum utilizing the ELISA System (BioTek Instruments, Inc., Winooski, VT, USA). The system comprised a microplate washer (model ELx50, BioTek Instruments, Inc., Winooski, VT, USA), an ELISA plate reader (model ELx800, BioTek Instruments, Inc., Winooski, VT, USA), and computer software (KCJunior, v1.41.3, BioTek Instruments, Inc., Winooski, VT, USA). Kits for Human Adiponectin High Sensitivity (Cat. No.: RD 191023100, BioVendor, LLC, Asheville, NC, USA), Human Resistin (Cat. No.: RD 191016100, BioVendor, LLC, Asheville, NC, USA), and Human Chemerin (Cat. No.: RD 191136200R, BioVendor, LLC, Asheville, NC, USA) were obtained from BioVendor Research and Diagnostics Products (Brno, Czech Republic). The Human Apelin Enzyme-Linked Immunosorbent Assay Kit was supplied by Cloud-Clone Corp. (Serial No.: 3BF65DA1A4) in Wuhan, Hubei, China. Standard curves were established using the reference standards provided with the kits and employed to ascertain the respective analyte concentrations for each sample. The minimum detection thresholds for individual analytes were as follows: adiponectin—1 ng/mL; resistin and chemerin—0.1 ng/mL; and apelin—0.2 pg/mL.

Cytokine concentrations were measured in blinded serum samples using the Bio-Plex 200 System from Bio-Rad and the Bio-Plex Pro Human Cytokine Panel 27-Plex (M500KCAF0Y, Hercules, CA, USA). Representative assay performance characteristics were as follows: specificity, analyte cross-reactivity < 10%, intra-assay precision % CV (coefficient of variation) < 15%, accuracy, percent recovery 70–130%. The Bio-Plex Suspension Array System included fluorescently labeled micro-spheres and instrumentation licensed to Bio-Rad Laboratories, Inc. by the Luminex Corporation (Austin, TX, USA). Standard curves were generated using the reference standards supplied with the kits and used to determine respective analyte concentrations for each sample.

### 2.4. Body-Mass Parameter Analysis

Body-mass parameters were evaluated using the InBody S10 body-composition analyzer (Biospace, Cerritos, CA, USA), which employs bioelectrical impedance spectroscopy to provide precise, efficient measurements [26]. The InBody S10 conforms to ISO 9001:2015 [27], ISO 13485:2016 [28], as well as medical standards EN60601-1 [29], EN60601-1-2 [30], and the CE MDD (Directive 93/42/EEC) [31]. To ensure high precision and reproducibility of bioimpedance analysis, a unified measurement protocol was applied throughout the study. Before assessment, participants were instructed to fast for at least three hours and to avoid alcohol intake for a minimum of twelve hours. They were also advised not to use medications that could alter fluid balance. All measurements were performed after the removal of heavy garments, metallic objects, and jewelry. Participants remained in an upright position for at least five minutes before the procedure. Demographic and anthropometric data, including age, sex, height, and body weight, were recorded before each measurement. Assessments were conducted at standardized time points to minimize variability related to circadian fluctuations. To verify measurement reliability, twelve repeated measurements were performed on the same individual under identical conditions. The coefficients of variation for all evaluated parameters remained below 3%, confirming the high repeatability of the method.

### 2.5. Statistical Analysis

The distribution of variables was assessed using the Shapiro–Wilk test, which indicated that the analyzed data did not follow a normal distribution. Therefore, non-parametric statistical methods were applied in further analyses. Quantitative variables were presented as medians with minimum and maximum values (min–max). Additionally, to provide a more comprehensive description of the data, mean values and standard deviations were included in the tables. Changes in clinical and biochemical parameters between two measurement points, i.e., before intervention (B-WRP) and post-intervention (P-WRP), were evaluated using Wilcoxon’s signed-rank test. Between-group differences were assessed using the Mann–Whitney U test. For statistically significant results, effect size (r) was also calculated based on the test statistic and sample size. The interpretation of effect size values was as follows: r < 0.10—trivial effect; 0.10 ≤ r < 0.30—small effect; 0.30 ≤ r < 0.50—medium effect; r ≥ 0.50—large effect; r ≥ 0.80—very large effect. Additionally, Spearman’s rank correlation analysis was performed to assess relationships between body-composition indices, physiological parameters, and cytokine concentrations measured in serum. Correlation matrices were additionally visualized using heatmaps with hierarchical clustering (dendrograms) to facilitate the identification of related patterns and groupings. A *p*-value < 0.05 was considered statistically significant. All statistical analyses were performed using Statistica 13.3 (version 13.3, TIBCO Software Inc., San Ramon, CA, USA, 2017) and the R environment with RStudio software (version 2026.01.1 Build 403).

## 3. Results

### 3.1. Anthropometry, Lipid, Glucose, and Oxidative Stress Parameters

Table 1, Table 2, Table 3 and Table 4 present comparative analyses of anthropometric, glucose metabolism, lipid, and oxidative stress parameters before and after the weight-reduction program, respectively.

Within-group analyses showed significant reductions in body weight, BMI, and WC in both women and men following the weight-reduction program. In women, body weight decreased from 92.3 kg to 84.75 (*p* < 0.001), with a very large effect size (r = 0.875). In men, body weight decreased from 107.5 kg to 100.0 (*p* = 0.002), also with a very large effect size (r = 0.883). Similar significant reductions were observed for BMI in both women (*p* < 0.001, r = 0.875) and men (*p* = 0.002, r = 0.883), as well as for WC (<0.001; women: r = 0.869; men: *p* = 0.002, r = 0.883).

Significant reductions were also observed in body fat parameters. VFA decreased markedly in women from 146.35 cm^2^ to 124.0 (*p* < 0.001, r = 0.857), and in men from 146.2 to 131.7 (*p* = 0.003, r = 0.687). BFM also decreased significantly in both sexes: in women from 40.25 kg to 36.5 (*p* = 0.001, r = 0.660) and in men from 37.25 kg to 32.95 (*p* = 0.007, r = 0.779). PBF decreased significantly in women—from 43.5% to 39.4 (*p* = 0.025, r = 0.457)—and in men from 34.55% to 32.3 (*p* = 0.028, r = 0.693).

Regarding lean components, significant decreases in FFM, SMM, and TBW were observed in women. FFM decreased from 53.9 kg to 51.2 (*p* ≤ 0.001, r = 0.709). SMM decreased from 28.95 kg to 27.3 (*p* ≤ 0.001, r = 0.683). TBW decreased from 39.5 L to 38.4 (*p* ≤ 0.001, r = 0.842). In men, reductions in FFM, SMM, and TBW did not reach statistical significance (*p*= 0.059), although effect sizes remained medium to large (r = 0.59).

Between-group comparisons after the intervention showed that men maintained significantly higher values of FFM, SMM, and TBW (all *p* < 0.001), corresponding to large effect sizes. Men also retained significantly higher body weight (*p* = 0.016) and WC (*p* = 0.038), whereas PBF remained significantly lower in men (*p* = 0.025).

Before the weight-reduction program, HOMA-IR was significantly higher in men than in women, as indicated by the median values 3.95 vs. 2.94 (*p* = 0.038), corresponding to a medium effect size. No sex-related differences were observed in fasting glucose or insulin concentrations. In contrast, HbA1c was significantly higher in men both before (*p* = 0.016) and after the intervention (*p* = 0.010). After the weight-reduction program, the sex-related difference in HOMA-IR was no longer significant (*p* = 0.745).

Within-group comparisons demonstrated a significant decrease in glucose levels in both sexes. In women, glucose decreased from 114.5 mg/dL to 90.5 (*p* < 0.001), with a large effect size (r = 0.729). In men, glucose decreased from 124.5 to 94.5 (*p* = 0.002), with a very large effect size (r = 0.883).

HOMA-IR also decreased significantly in both groups: in women, from 2.94 to 2.09 (*p* = 0.009, r = 0.545) and in men from 3.95 to 3.05 (*p* = 0.023, r = 0.657). HbA1c decreased significantly in women: from 5.26% to 5.01 (*p* = 0.001, r = 0.688, large effect). In men, the reduction was not statistically significant (*p* = 0.131, r = 0.456, medium effect).

Non-parametric analysis showed no significant changes in total cholesterol concentration. In women, values were 232.5 mg/dL before the intervention and 226.45 after the intervention (*p* = 0.601, r = 0.117). In men, total cholesterol also did not change significantly: from 206.0 to 189.0 (*p* = 0.48, r = 0.204). A significant decrease in triglycerides was observed in women, with values decreasing from 119.0 mg/dL to 99.0 (*p* = 0.03), corresponding to a medium effect size (r = 0.453). In men, the change was not significant (*p* = 0.583, r = 0.159, small effect). HDL-C concentration increased significantly in men, from 50.0 mg/dL to 55.5 (*p* = 0.019), with a large effect size (r = 0.679). In women, the increase in HDL-C represented a non-significant trend, from 55.0 to 57.5 (*p* = 0.086, r = 0.366, medium effect). There was no significant change in LDL-C concentration in either group (in women: *p* = 0.879, r = 0.036, trivial effect), (in men: *p* = 0.214, r = 0.359, medium effect). The new-AIP index improved significantly in both groups. In women, it decreased from 1.04 to 0.75 (*p* = 0.011, r = 0.519, large effect). In men, AIP also decreased significantly, from 1.0 to 0.85 (*p* = 0.023, r = 0.657, large effect).

Non-parametric analysis revealed a significant reduction in both MDA concentration and OSI values in both groups. In women, MDA concentration decreased from 2.86 µmol/L to 2.12 µmol/L (*p* = 0.013), accompanied by a large effect size (r = 0.500). In men, a similar and also significant reduction in median MDA was observed, from 3.46 to 2.20 (*p* = 0.005), with a very large effect size (r = 0.820). Sex-related differences approached statistical significance before the intervention (*p* = 0.062) and were non-significant after the intervention (*p* = 0.482). A similar pattern was observed for OSI. In women, median OSI decreased from 4.26 before the intervention to 1.73 afterwards (*p* < 0.001), with a very large effect size (r = 0.834). In men, OSI decreased from 4.12 to 1.09 (*p* = 0.034), with a large effect size (r = 0.611). Sex-related differences were non-significant both before (*p* = 0.719) and after the intervention (*p* = 0.127).

### 3.2. Cytokine Concentrations

Cytokine levels are presented in Table 5.

Significant reductions were observed in several inflammatory cytokines. IL-1β decreased significantly in women, from 4.09 pg/mL to 3.29 (*p* = 0.046), with a medium effect (r = 0.447), while in men the reduction did not reach statistical significance, although the effect size suggested a large effect (r = 0.532). IL-1ra decreased significantly in both women—from 61.68 to 51.58 (*p* = 0.017, r = 0.559, large)—and men—from 52.5 to 39.11 (*p* = 0.026, r = 0.566, large). IL-6 showed a marked decrease in both sexes: in women from 13.8 to 9.94 (*p* = 0.009, r = 0.600, large) and in men from 9.44 to 5.13 (*p* = 0.007, r = 0.854, very large). A significant reduction was also found for IL-7 in men—from 28.47 to 21.15 (*p* = 0.041, r = 0.589, large)—while the change in women was non-significant. TNF-α decreased significantly in both women—from 20.0 to 16.15 (*p* = 0.018, r = 0.545, large)—and men—from 27.1 to 21.1 (*p* = 0.019, r = 0.741, large, approaching very large). In contrast, cytokines IL-4, IL-5, IL-8, IL-12, and IL-17 showed no statistically significant changes in either sex, and effect sizes ranged from trivial to medium (r = 0.023–0.365), indicating only minor or clinically negligible shifts.

### 3.3. Correlations

**Figure 1:**
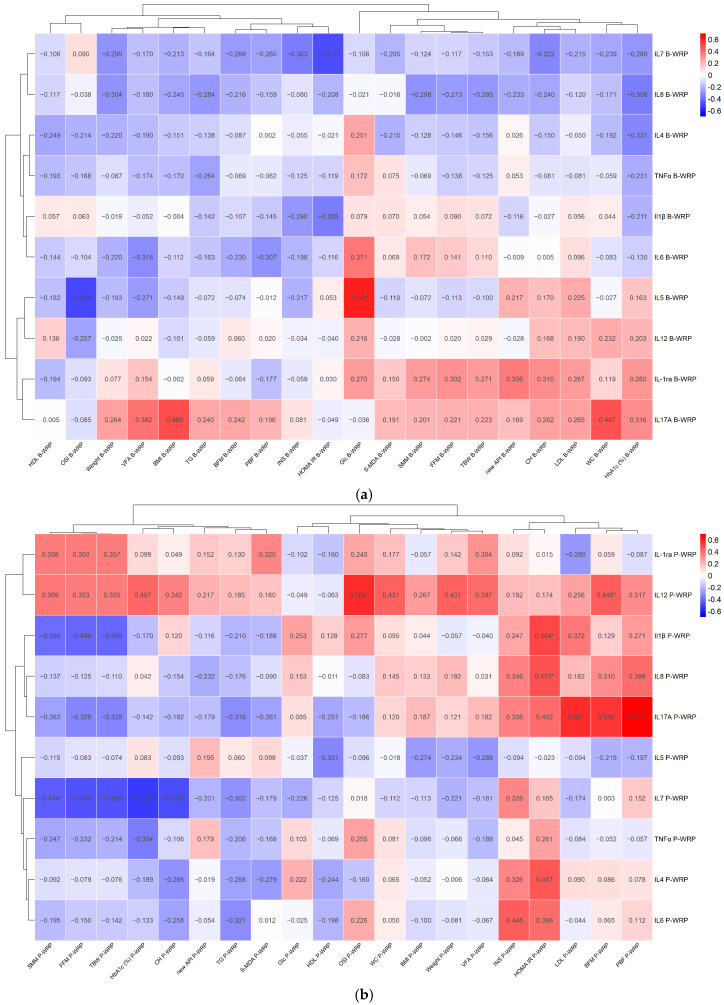
Correlation matrix between cytokine levels (IL-1β, IL-1ra, IL-4, IL-5, IL-6, IL-7, IL-8, IL-12, IL-17A, TNF-α) and metabolic or body-composition parameters (MDA, HDL, CH, SMM, PBF, VFA, HOMA-IR, Glc, WC, weight) in females, shown before (**a**) and after (**b**) WRP. Positive and negative numbers represent positive (red) and negative (blue) correlations. Values closer to 1 or −1 indicate stronger associations. *—significant *p*-value.

**Figure 2:**
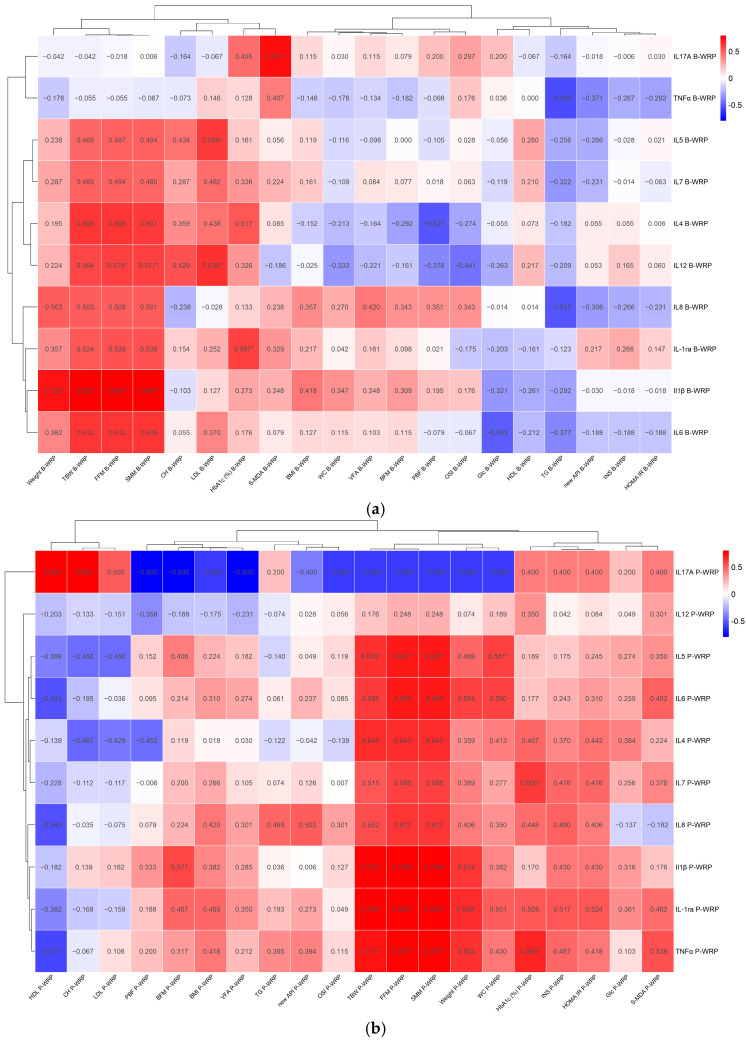
Correlation matrix between cytokine levels (IL-1β, IL-1ra, IL-4, IL-5, IL-6, IL-7, IL-8, IL-12, IL-17A, TNF-α) and metabolic or body-composition parameters (MDA, HDL, CH, SMM, PBF, VFA, HOMA-IR, Glc, WC, weight) in males, shown before (**a**) and after (**b**) WRP. Positive and negative numbers represent positive (red) and negative (blue) correlations. Values closer to 1 or −1 indicate stronger associations. *—significant *p*-value.

**Figure 3:**
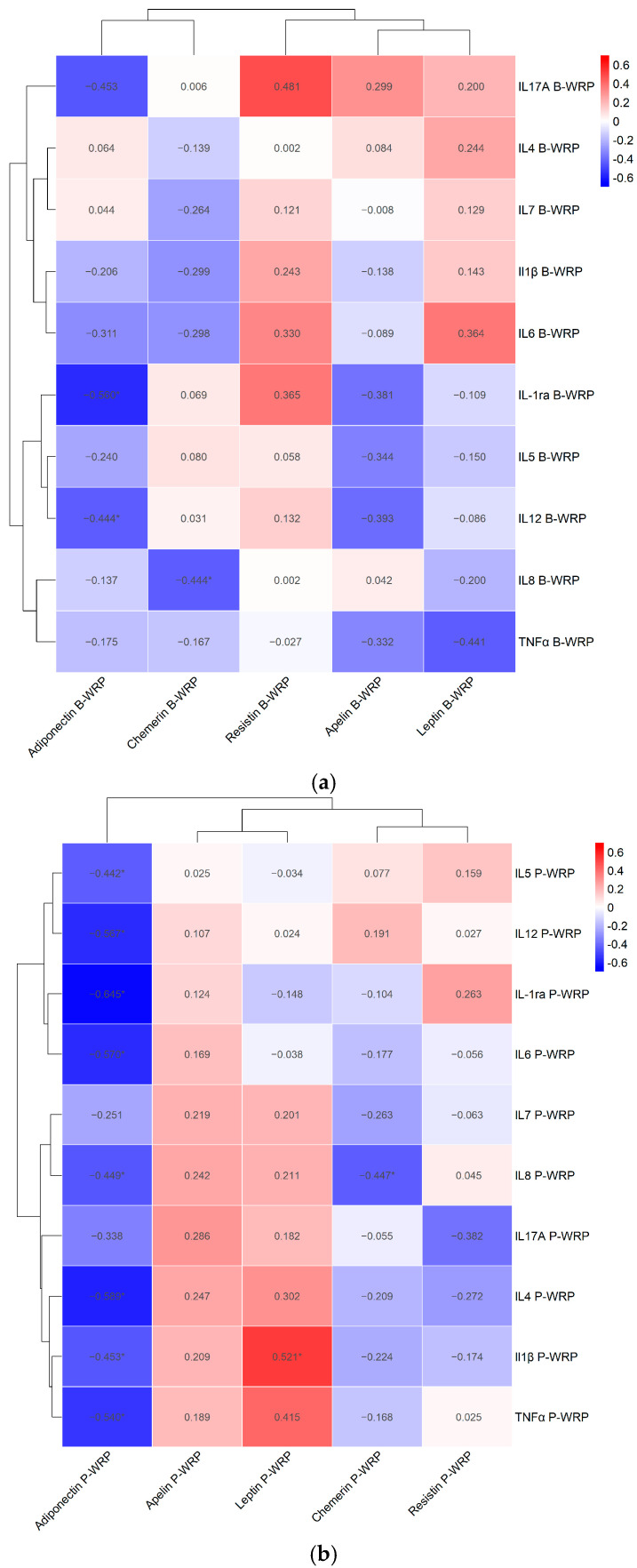
Correlation matrix between cytokine levels (IL-1β, IL-1ra, IL-4, IL-5, IL-6, IL-7, IL-8, IL-12, IL-17A, TNF-α) and adipokine parameters (leptin, apelin, resistin, chemerin, adiponectin) in females, shown before (**a**) and after (**b**) WRP. Positive and negative numbers represent positive (red) and negative (blue) correlations. Values closer to 1 or −1 indicate stronger associations. *—significant *p*-value.

**Figure 4:**
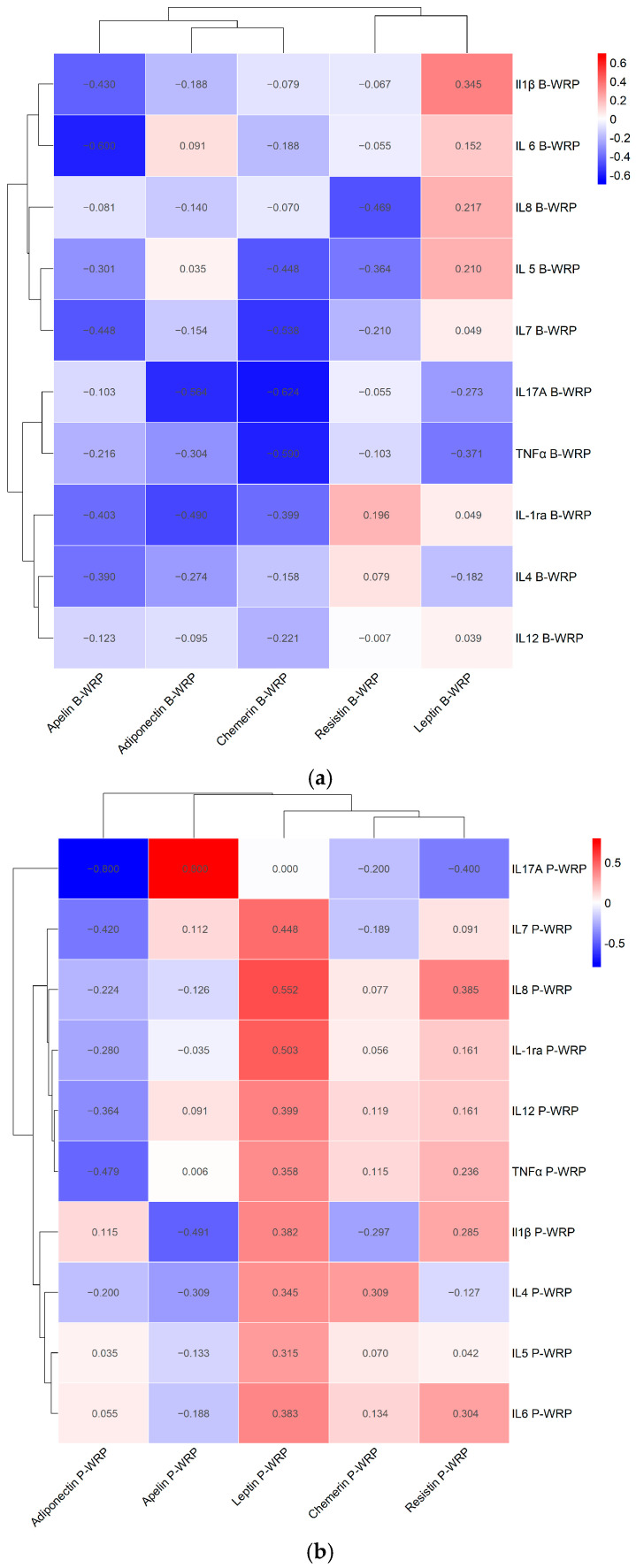
Correlation matrix between cytokine levels (IL-1β, IL-1ra, IL-4, IL-5, IL-6, IL-7, IL-8, IL-12, IL-17A, TNF-α) and adipokine parameters (leptin, apelin, resistin, chemerin, adiponectin) in males, shown before (**a**) and after (**b**) WRP. Positive and negative numbers represent positive (red) and negative (blue) correlations. Values closer to 1 or −1 indicate stronger associations.

The correlation structures between cytokine concentrations and selected anthropometric, body composition, glycemic, lipid, oxidative, and adipokine parameters—separately in women and men—before and after the weight-reduction program are presented in Figure 1, Figure 2, Figure 3 and Figure 4.

Spearman’s correlation analysis revealed sex-related differences in the structure of associations between cytokines and anthropometric, metabolic, oxidative, and adipokine variables, both before and after the intervention. Before weight reduction, the cytokine profile in women showed relatively moderate associations with glycemic, lipid, and adipokine parameters. In this group, the strongest positive coefficients were observed for IL-5 with glucose (r = 0.594) and for IL-17A with BMI (r = 0.489), waist circumference (r = 0.467), and visceral fat area (r = 0.382). Adiponectin showed negative associations with several cytokines, including IL-1ra (r = −0.560), IL-12 (r = −0.444), and IL-17A (r = −0.453).

In men, before weight reduction, cytokines were more strongly associated with lean body composition. The highest positive coefficients were observed between IL-1β and fat-free mass, skeletal-muscle mass, and total body water (r = 0.845, 0.845, and 0.830, respectively), as well as between IL-12 and fat-free mass, skeletal-muscle mass, and LDL-C (r = 0.579, 0.577, and 0.634, respectively). In the adipokine panel, chemerin showed negative associations with IL-7, IL-17A, and TNF-α (r = −0.538, −0.624, and −0.590, respectively).

After weight reduction, the correlation structure in women shifted toward insulin resistance, body composition, and adipokine-related associations. IL-1β showed a positive association with HOMA-IR (r = 0.504), while several cytokines were inversely associated with lean body-mass parameters, particularly IL-7 with HbA1c, fat-free mass, skeletal-muscle mass, and total body water (r = −0.529, −0.464, −0.444, and −0.465, respectively). IL-17A was positively associated with body fat mass, percent body fat, and LDL-C (r = 0.536, 0.687, and 0.561, respectively). In addition, adiponectin displayed broad negative associations with multiple cytokines after WRP, including IL-1ra, IL-4, IL-6, IL-8, IL-12, and TNF-α.

In men after weight reduction, associations with lean body mass became even more pronounced. Strong positive coefficients were observed between IL-1β and fat-free mass, skeletal-muscle mass, and total body water (r = 0.786, 0.786, and 0.762, respectively), between IL-1ra and the same parameters (r = 0.842, 0.842, and 0.782), and between TNF-α and fat-free mass, skeletal-muscle mass, total body water, and HbA1c (r = 0.767, 0.767, 0.717, and 0.663, respectively). IL-17A showed an opposite pattern, with negative associations with body weight, BMI, waist circumference, visceral fat area, body fat mass, percent body fat, and lean mass parameters, and positive associations with total cholesterol and HDL-C (r = 0.800 for both). In the adipokine analysis, leptin showed positive associations with several cytokines, including IL-1ra, IL-7, IL-8, and IL-12.

Overall, the correlation analysis suggests that weight reduction was accompanied by a sex-specific reorganization of the immunometabolic network. In women, post-intervention associations were more strongly linked to insulin resistance, adiposity, and adiponectin-related regulation, whereas in men, the cytokine profile remained more closely connected to lean body mass and selected lipid parameters.

## 4. Discussion

The present study shows that a 6-month lifestyle-based weight-reduction program in adults with overweight or obesity and insulin resistance was associated with improvements in several metabolic domains, including body weight, abdominal adiposity, glucose homeostasis, new-AIP-based atherogenic risk, and oxidative balance. This metabolic improvement was accompanied by selective rather than uniform changes in the circulating cytokine profile. The most consistent common response in both women and men was a decrease in IL-1ra, IL-6, and TNF-α. In sex-stratified analyses, IL-1β decreased significantly only in women, whereas IL-7 decreased significantly only in men. These findings suggest partial attenuation of obesity-related low-grade inflammation after lifestyle-induced weight reduction. However, given the before-and-after design, the relatively small sex-stratified subgroups, and the number of cytokine endpoints analyzed, these sex-related cytokine patterns should be interpreted cautiously and regarded as hypothesis-generating rather than definitive evidence of sex-specific causal effects [8,15]. The decrease in IL-6 in both sexes is biologically plausible and is consistent with the evidence, indicating that clinically meaningful weight loss, particularly above the 5% threshold, is more likely to reduce circulating IL-6 in adults with obesity [15]. Nevertheless, IL-6 should be interpreted in a context-dependent manner. In obesity, chronically elevated IL-6 may reflect adipose-tissue inflammation and metabolic stress, whereas during physical activity, IL-6 can also act as a myokine involved in substrate mobilization and anti-inflammatory signaling [32,33]. In the present study, the decrease in IL-6 was accompanied by reductions in waist circumference, visceral fat area, HOMA-IR, and OSI. This supports the interpretation that the intervention was accompanied by a lower chronic inflammatory and oxidative burden, although the present design does not allow the decrease in IL-6 to be attributed exclusively to weight reduction itself. The decrease in TNF-α observed in both women and men is also clinically relevant because TNF-α is closely linked to obesity-associated insulin resistance and impaired lipid metabolism. Experimental and translational studies suggest that TNF-α contributes to disrupted insulin signaling and dyslipidemia, although clinical intervention studies have not consistently shown a strong decrease in circulating TNF-α following lifestyle-induced weight loss [32,34]. A recent meta-analysis of dietary weight-loss trials reported a clearer effect for IL-6 than for TNF-α [15]. In this context, the observed reduction in TNF-α may indicate that a multimodal intervention combining caloric restriction, behavioral support, and regular physical activity can attenuate systemic inflammatory activation associated with insulin-resistant obesity. This finding should still be interpreted at the group level, because cytokine concentrations show substantial biological variability and are not yet established as routine markers for monitoring individual clinical responses to obesity treatment [15,32]. The IL-1-related pathway is another relevant component of the inflammatory response. IL-1 family mediators are involved in the relationship between nutrient excess, innate immune activation, and insulin resistance [34,35]. In the present study, IL-1ra decreased in both sexes, whereas IL-1β decreased significantly only in women. IL-1ra is often interpreted as a compensatory anti-inflammatory response to enhanced IL-1 pathway activation; therefore, its decrease after the intervention may reflect a lower need for counter-regulation as metabolic stress subsides. The decrease in IL-1β observed in women may suggest attenuation of inflammasome-related signaling, but this interpretation should remain cautious because the finding was obtained in a subgroup analysis, and the *p*-value was close to the conventional threshold for statistical significance. Thus, the IL-1β result should be considered exploratory and should be confirmed in larger, sex-balanced cohorts. A major strength of the study is that inflammatory data were interpreted together with detailed body-composition measurements. Both women and men achieved significant reductions in body weight, BMI, waist circumference, visceral fat area, body fat mass, and body fat percentage. However, women also showed significant decreases in fat-free mass, skeletal-muscle mass, and total body water, whereas in men, these changes followed the same direction but did not reach statistical significance. This finding is important because it shows that the metabolic benefits of weight loss may coexist with the loss of lean mass, skeletal-muscle mass, and total body water. Our previous studies using the same weight-reduction model showed that improvement in carbohydrate metabolism may coexist with the unwanted loss of lean tissue and body water, especially in metabolically vulnerable subgroups [17,19,20]. The present results support the clinical relevance of evaluating not only the magnitude of weight loss, but also the biological quality of weight loss, including the preservation of lean mass and body-water compartments.

The sex-stratified character of the analysis should be interpreted with appropriate caution. Current evidence indicates that women and men differ in fat distribution, insulin sensitivity, adipose-tissue inflammation, adipocytokine production, and immune responses in obesity [12,13]. In our cohort, broad baseline differences across all cytokines were not observed, but several post-intervention response patterns differed between women and men. Women showed decreases in IL-1β, IL-1ra, IL-6, and TNF-α, whereas men showed decreases in IL-1ra, IL-6, IL-7, and TNF-α. In addition, TNF-α remained higher in women than in men at both time points. These findings may indicate different inflammatory set points and different modes of immunometabolic adaptation during weight reduction. However, a statistically significant change in one subgroup and a non-significant change in another subgroup should not by itself be interpreted as proof of a true sex-by-time interaction. Therefore, the present results are best viewed as sex-stratified patterns that require confirmation using formal interaction testing and larger balanced samples.

The oxidative-stress results reinforce the interpretation that the intervention was associated with improved systemic metabolic health in both sexes. MDA and OSI decreased significantly in both women and men, indicating reduced lipid peroxidation and overall oxidant burden after the program. These findings are consistent with our earlier reports showing that weight reduction in obesity is accompanied by improvement in serum and erythrocyte redox balance, although the exact redox response depends on glycemic status and on the type of tissue lost during intervention [19,20]. In the present study, the simultaneous improvements in oxidative-stress markers and selected cytokines support the concept that the benefits of lifestyle intervention may involve interconnected metabolic, inflammatory, and redox pathways rather than anthropometric change alone. Cardiometabolic improvement after weight reduction also showed sex-stratified biochemical patterns. In women, triacylglycerols and new-AIP improved significantly, whereas in men, HDL-C increased, and new-AIP also improved. Thus, both sexes showed a more favorable lipid-related risk profile after intervention, but not through identical biochemical changes. This is consistent with our previous observation that lipid improvement during weight reduction depends on metabolic context rather than body-weight loss alone [18,23]. From a clinical perspective, these findings support the use of integrated monitoring that includes glucose–insulin homeostasis, modified atherogenic risk, inflammatory markers, oxidative stress, and body composition, rather than relying only on body weight or BMI.

The correlation analysis provides an additional exploratory layer of biological context, but it should not be overinterpreted. Because multiple correlations were examined in a relatively small cohort, especially in the male subgroup, these analyses should be considered hypothesis-generating. After weight reduction, women showed broader inverse associations between adiponectin and several cytokines, which may reflect a more anti-inflammatory and insulin-sensitive regulatory profile. In men, cytokine associations remained more closely related to fat-free mass, skeletal-muscle mass, and total body water. These patterns are biologically plausible, but they do not establish causality. They suggest that weight reduction may reorganize inflammatory–metabolic relationships in a sex-related manner, and they justify further investigation in larger controlled studies with balanced sex representation, longer follow-up, objective adherence monitoring, and statistical models designed to evaluate sex-by-time interaction effects.

### Study Limitations

This study has several limitations that should be considered when interpreting the findings. First, it was a single-center, before-and-after lifestyle intervention study that included only adults with overweight or obesity and insulin resistance who completed the 6-month weight-reduction program and had complete anthropometric, body-composition, biochemical, oxidative-stress, adipokine, and cytokine data at both time points. Therefore, the findings may not be generalizable to all individuals with obesity, to patients without insulin resistance, or to populations treated in other clinical settings. Second, the study did not include a separate non-intervention, weight-stable, or healthy control group. The baseline values obtained before the weight-reduction program served as the reference point for the post-intervention assessment, allowing for within-subject evaluation of metabolic, inflammatory, and body-composition adaptations. This approach is clinically meaningful in view of the substantial inter-individual variability in biochemical and inflammatory parameters among people with obesity and insulin resistance. However, the absence of a separate control group limits causal interpretation. We cannot fully exclude the contribution of temporal variability, regression toward the mean, or other uncontrolled lifestyle factors to some of the observed changes. Third, the final sample size was relatively small and sex-imbalanced, particularly in the male subgroup (*n* = 12). The sample-size calculation was based on the expected weight-reduction outcome rather than on cytokine endpoints, sex-stratified cytokine responses, or sex-by-time interaction effects. Therefore, the study may be underpowered for extensive subgroup analyses and for definitive assessment of sex-related differences in immunometabolic adaptation. Cytokine findings close to the conventional threshold of statistical significance should, therefore, be interpreted cautiously and regarded as hypothesis-generating. Fourth, the strict inclusion and exclusion criteria, the requirement for HOMA-IR ≥ 2, the exclusion of participants receiving treatment affecting glucose or lipid metabolism, completion of the 6-month follow-up, and the requirement for complete laboratory and cytokine datasets may have introduced selection bias. Some participants did not complete the 6-month weight-reduction program because they discontinued follow-up visits, did not adhere to the dietary and physical-activity recommendations, were later found to meet exclusion criteria, or had incomplete data. As a result, the final cohort may have included participants with better adherence to the intervention, which may limit the external validity and generalizability of the results. Fifth, adherence to the intervention was monitored during regular follow-up visits using anthropometric measurements, participant interviews, and self-reported dietary and physical-activity diaries. These records were reviewed and used to provide individualized feedback and motivational support. Nevertheless, caloric intake was not assessed using weighed dietary records or standardized repeated dietary recalls, and physical activity was not objectively quantified using accelerometers, pedometers, or wearable devices. Therefore, inter-individual differences in actual adherence to diet and exercise could have influenced metabolic and inflammatory responses. Sixth, body composition was assessed using bioelectrical impedance analysis. Although measurements were performed according to a standardized protocol, and this method is practical for repeated clinical assessment, it is not a reference technique, such as dual-energy X-ray absorptiometry or magnetic resonance imaging. Hydration status and other physiological factors may affect estimates of fat-free mass, skeletal-muscle mass, and total body water. Seventh, female hormonal status, menopausal status, menstrual-cycle phase, and sex-hormone concentrations were not systematically assessed. These factors may influence fat distribution, insulin sensitivity, lipid metabolism, and cytokine secretion, and their absence may have contributed to variability in the female subgroup. Finally, the use of a multiplex cytokine panel in a relatively small cohort requires careful interpretation. Numerous comparisons across anthropometric, metabolic, oxidative-stress, adipokine, and cytokine variables increase the risk of type I error, particularly for marginal *p*-values. In addition, the correlation analyses were exploratory and should not be interpreted as evidence of causal relationships. The observed sex-stratified patterns, therefore, require confirmation in larger, controlled, sex-balanced studies with longer follow-up, objective adherence monitoring, hormonal profiling, and statistical approaches specifically designed to evaluate sex-by-time interaction effects and multiple-comparison control.

## 5. Conclusions

In summary, a 6-month individualized lifestyle-based weight-reduction program in adults with overweight/obesity and insulin resistance was associated with significant improvements in body weight, abdominal adiposity, glucose–insulin homeostasis, new-AIP-based atherogenic risk, and oxidative balance. These findings indicate broad metabolic benefits of the intervention, but they also show that the responses to weight reduction should not be evaluated solely on the basis of total body-weight loss or BMI.

The inflammatory response was selective rather than uniform. IL-1ra, IL-6, and TNF-α decreased in both women and men, supporting partial attenuation of obesity-related low-grade inflammation. In sex-stratified analyses, IL-1β decreased significantly only in women, whereas IL-7 decreased significantly only in men. However, these findings should be interpreted with caution due to the limited sample size, the number of comparisons performed, and the measurement of multiple cytokines in a relatively small group of patients.

The results also suggest that the type of body-composition changes accompanying weight reduction may differ between women and men. In both sexes, the intervention reduced obesity-related parameters, but in women, it was also accompanied by significant reductions in fat-free mass, skeletal-muscle mass, and total body water. This observation is clinically relevant because metabolically beneficial weight reduction may coexist with potentially unfavorable changes in body composition.

The findings show that the assessment of the effectiveness of weight reduction should include not only body-weight loss but also metabolic, inflammatory, oxidative, and body-composition parameters, analyzed separately in women and men. In clinical and research settings, treatment response should, therefore, be evaluated using not only body weight and BMI, but also visceral adiposity, lean mass, glucose–insulin homeostasis, atherogenic lipid risk, oxidative stress, and selected inflammatory markers. Larger controlled studies with a more balanced representation of women and men, longer follow-up, and statistical models designed to assess sex-by-time interactions are needed to confirm whether the observed patterns truly reflect sex-related differences in immunometabolic adaptation to weight reduction.

## Figures and Tables

**Table 1:** Anthropometry and body composition of participants of the study, divided by sex, before (B-WRP) and after (P-WRP) the weight-reduction program. Data are presented as median (Q1–Q3), where Q1 represents the 25th percentile and Q3 represents the 75th percentile, together with minimum–maximum values. Differences are considered significant if *p* < 0.05.

	Sex	Female (*n* = 24)	Male (*n* = 12)	*p* (F vs. M)
		median (Q1–Q3) min–max	median (Q1–Q3) min–max	
Weight [kg]	B-WRP	92.3 (86.95–115.25)	107.5 (96.5–129.5)	0.062 *
		76–139	86–158	
	P-WRP	84.75 (78.75–100)	100.0 (91–115.9)	0.016 *
		68–126	76–145	
*p* (B-WRP vs. P-WRP)		<0.001 *	0.002 *	
BMI [kg/m^2^]	B-WRP	36.38 (34.18–40.85)	35.19 (33.03–40.88)	0.704
		27.92–54.19	28.73–51.29	
	P-WRP	33.37 (29.89–35.9)	33.47 (30.91–36.66)	0.679
		25.91–50.54	25.39–47.89	
*p* (B-WRP vs. P-WRP)		<0.001 *	0.002 *	
WC [cm]	B-WRP	111.5 (103–120)	121.5 (112–132.5)	0.057
		91–134	98–139	
	P-WRP	99.0 (91.5–109.5)	109.0 (101.75–121)	0.038 *
		73–127	90–132	
*p* (B-WRP vs. P-WRP)		<0.001 *	0.002 *	
VFA [cm^2^]	B-WRP	146.35 (135.35–170.4)	146.2 (117.8–197.65)	0.960
		112–195.40	88.7–210.6	
	P-WRP	124.0 (97.5–143.1)	131.7 (97.15–167.95)	0.420
		73.70–168.60	63.6–181.9	
*p* (B-WRP vs. P-WRP)		<0.001 *	0.003 *	
BFM [kg]	B-WRP	40.25 (35.15–53.35)	37.75 (27.5–53.5)	0.327
		29.1–72.2	19.7–76.7	
	P-WRP	36.5 (29.2–42.6)	32.95 (26.3–46.5)	0.795
		18.8–76.8	16.4–65.5	
*p* (B-WRP vs. P-WRP)		0.001 *	0.007 *	
PBF [%]	B-WRP	43.5 (39.4–50.2)	34.55 (29.1–39.05)	0.001 *
		34.8–54.2	18.5–49.4	
	P-WRP	39.45 (33.5–48.1)	35.05 (30–36.8)	0.025 *
		26.3–53.8	21.5–43.6	
*p* (B-WRP vs. P-WRP)		0.025 *	0.028 *	
FFM [kg]	B-WRP	53.9 (49.8–57.4)	72.7 (64.05–78.6)	<0.001 *
		44.5–81.8	57.8–102.4	
	P-WRP	52.1 (47.2–54.6)	68.05 (60.8–74.4)	<0.001 *
		41.4–72.4	56.3–92.7	
*p* (B-WRP vs. P-WRP)		0.001 *	0.059	
SMM [kg]	B-WRP	29.85 (27.1–32.15)	41.35 (36.5–45.15)	<0.001 *
		24.3–47.0	31.6–57.0	
	P-WRP	28.7 (25.8–30.4)	38.7 (33.8–42)	<0.001 *
		22.9–40.7	30.9–51.4	
*p* (B-WRP vs. P-WRP)		0.001 *	0.059	
TBW [L]	B-WRP	39.5 (36.5–42.3)	53.5 (46.95–57.85)	<0.001 *
		32.7–59.9	42.5–76.0	
	P-WRP	38.4 (34.2–40.3)	50.05 (44.4–54.8)	<0.001 *
		30.2–53.1	41.6–68.7	
*p* (B-WRP vs. P-WRP)		<0.001 *	0.059	

Legend: B-WRP—before weight-reduction program; P-WRP—post-weight-reduction program; BMI—Body Mass Index; WC—waist circumference; VFA—visceral fat area; BFM—body fat mass; PBF—percentage of body fat; FFM—fat-free mass; SMM—skeletal-muscle mass; TBW—total body water; *—significant *p*-value.

**Table 2:** Glycemia and insulin resistance parameters of participants of the study, divided by sex, before (B-WRP) and after (P-WRP) the weight-reduction program. Data are presented as median (Q1–Q3), where Q1 represents the 25th percentile and Q3 represents the 75th percentile, together with minimum–maximum values. Differences are considered significant if *p* < 0.05.

	Sex	Female (*n* = 24)	Male (*n* = 12)	*p* (F vs. M)
		median (Q1–Q3) min–max	median (Q1–Q3) min–max	
Glucose [mg/dL]	B-WRP	114.5 (98–128.5)	124.5 (111.5–161)	0.137
		80–330	93–175	
	P-WRP	90.5 (76–100)	94.5 (88–102)	0.416
		59–150	65–104	
*p* (B-WRP vs. P-WRP)		<0.001 *	0.002 *	
Insulin [µIU/mL]	B-WRP	11.6 (9.39–15.2)	13.32 (9.3–26.22)	0.327
		7.05–37.31	8.66–47.16	
	P-WRP	10.37 (8.6–13.48)	12.4 (6.88–22.75)	0.851
		3.19–37.79	2.92–38.34	
*p* (B-WRP vs. P-WRP)		0.212	0.071	
HOMA-IR	B-WRP	2.94 (2.5–4.27)	3.95 (3.31–7.42)	0.038 *
		2.11–15.18	2.47–15.26	
	P-WRP	2.09 (1.85–3.47)	3.05 (1.31–5.48)	0.745
		0.66–8.86	0.66–9.84	
*p* (B-WRP vs. P-WRP)		0.009 *	0.023 *	
HbA1c [%]	B-WRP	5.26 (5.12–5.54)	5.92 (5.34–6.35)	0.016 *
		4.45–6.31	4.89–6.68	
	P-WRP	5.01–5.39	5.29–6.14	0.010 *
		3.60–6.00	4.87–6.95	
*p* (B-WRP vs. P-WRP)		0.001 *	0.131	

Legend: B-WRP—before weight-reduction program; P-WRP—post-weight-reduction program; HOMA-IR—Homeostatic Model Assessment of Insulin Resistance; HbA1c—glycated hemoglobin A_1c_; *—significant *p*-value.

**Table 3:** Lipid profile parameters of participants of the study, divided by sex, before (B-WRP) and after (P-WRP) the weight-reduction program. Data are presented as median (Q1–Q3), where Q1 represents the 25th percentile and Q3 represents the 75th percentile, together with minimum–maximum values. Differences are considered significant if *p* < 0.05.

	Sex	Female (*n* = 24)	Male (*n* = 12)	*p* (F vs. M)
		median (Q1–Q3) min–max	median (Q1–Q3) min–max	
CH [mg/dL]	B-WRP	232.5 (204.5–271)	206.0 (182–243)	0.166
		140–377	136–268	
	P-WRP	226.45 (208–241)	189.0 (168.15–235.5)	0.044 *
		175–311	155–258	
*p* (B-WRP vs. P-WRP)		0.601	0.48	
TG [mg/dL]	B-WRP	119.0 (85–187)	97.5 (82–143.5)	0.526
		51–226	58–227	
	P-WRP	99.0 (80–124)	106.3 (73.5–139)	0.668
		53–226	42–207	
*p* (B-WRP vs. P-WRP)		0.03 *	0.583	
HDL-C [mg/dL]	B-WRP	55.0 (52–64)	50.0 (43.5–60.5)	0.092
		44–78	31–70	
	P-WRP	57.5 (54–68)	55.5 (47.15–67)	0.245
		40–85	41.9–69	
*p* (B-WRP vs. P-WRP)		0.086	0.019 *	
LDL-C [mg/dL]	B-WRP	139 (131–179)	137.5 (103.5–157.5)	0.381
		71–216	84–187	
	P-WRP	146 (129–155)	104 (94–147)	0.076
		100–200	104.00 (87–171)	
*p* (B-WRP vs. P-WRP)		0.879	0.214	
new-AIP	B-WRP	1.04 (0.56–1.15)	1 (0.71–1.27)	0.562
		0.33–1.72	0.34–1.74	
	P-WRP	0.75 (0.52–0.96)	0.9 (0.55–1.16)	0.456
		0.38–1.46	0.26–1.29	
*p* (B-WRP vs. P-WRP)		0.011 *	0.023 *	

Legend: B-WRP—before weight-reduction program; P-WRP—post-weight-reduction program; CH—total cholesterol; TG—triacylglycerols; HDL-C—cholesterol in high-density lipoproteins; LDL-C—cholesterol in low-density lipoproteins; new-AIP—modified Atherogenic Index of Plasma; *—significant *p*-value.

**Table 4:** Oxidative stress parameters of participants of the study, divided by sex, before (B-WRP) and after (P-WRP) the weight-reduction program. Data are presented as median (Q1–Q3), where Q1 represents the 25th percentile and Q3 represents the 75th percentile, together with minimum–maximum values. Differences are considered significant if *p* < 0.05.

	Sex	Female (*n* = 24)	Male (*n* = 12)	*p* (F vs. M)
		median (Q1–Q3) min–max	median (Q1–Q3) min–max	
MDA [µmol/L]	B-WRP	2.86 (2.2–3.62)	3.46 (3.05–4.09)	0.062
		1.07–4.98	1.84–6.51	
	P-WRP	2.12 (1.6–2.64)	2.2 (1.95–2.75)	0.482
		0.95–4.36	1.10–3.50	
*p* (B-WRP vs. P-WRP)		0.013 *	0.005 *	
OSI	B-WRP	4.26 (2.82–9.16)	4.12 (2.44–9.1)	0.719
		1.39–18.87	1.15–12.16	
	P-WRP	1.73 (1.12–2.2)	1.09 (0.64–2.04)	0.127
		0.75–3.59	0.11–11.92	
*p* (B-WRP vs. P-WRP)		<0.001 *	0.034 *	

Legend: B-WRP—before weight-reduction program; P-WRP—post-weight-reduction program; MDA—malondialdehyde; OSI—Oxidative Stress Index; *—significant *p*-value.

**Table 5:** Cytokine levels of participants of the study, divided by sex, before (B-WRP) and after (P-WRP) the weight-reduction program. Data are presented as median (Q1–Q3), where Q1 represents the 25th percentile and Q3 represents the 75th percentile, together with minimum–maximum values. Differences are considered significant if *p* < 0.05.

	Sex	Female (*n* = 24)	Male (*n* = 12)	*p* (F vs. M)
		median (Q1–Q3) min–max	median (Q1–Q3) min–max	
IL-1β	B-WRP	4.09 (2.81–5.85)	3.43 (1.96–4.37)	0.393
		1.12–19.32	0.88–13.25	
	P-WRP	3.29 (2.7–3.85)	2.57 (2.12–4.23)	0.350
		1.4–8.62	0.85–9.53	
*p* (B-WRP vs. P-WRP)		0.046 *	0.093	
IL-1ra	B-WRP	61.63 (38.86–84.64)	63.78 (43.6–81.46)	0.889
		19.78–257.63	17.3–228.76	
	P-WRP	51.68 (42.23–62.49)	38.97 (31.41–61.69)	0.267
		9.6–139.25	2.71–133.62	
*p* (B-WRP vs. P-WRP)		0.018 *	0.049 *	
IL-4	B-WRP	4.09 (3.39–4.83)	3.68 (3.25–3.95)	0.221
		2.54–7.57)	1.51–5.88	
	P-WRP	3.68 (3.09–4.69)	3.25 (2.83–3.54)	0.176
		2.2–5.6	1.29–5.62	
*p* (B-WRP vs. P-WRP)		0.426	0.203	
IL-5	B-WRP	12.97 (8.37–17.6)	10.1 (7.62–16.15)	0.54
		0.96–43.54	5.57–43.32	
	P-WRP	11.67 (10.25–15.95)	10.42 (6.93–15.68)	0.344
		11.67 (3.26–24.58)	10.42 (2.71–33.27	
*p* (B-WRP vs. P-WRP)		0.273	0.272	
IL-6	B-WRP	13.8 (10.12–19.74)	11.22 (8.94–15.66)	0.745
		3.66–93.99	5.13–55.95	
	P-WRP	8.94 (7.29–14.33)	7.78 (4.32–13.09)	0.266
		3.41–39.88	1.98–43.9	
*p* (B-WRP vs. P-WRP)		0.009 *	0.007 *	
IL-7	B-WRP	28.41 (22.35–41.4)	24.87 (17.48–40.46)	0.436
		11.14–99.48	9.9–52.02	
	P-WRP	21.52 (16.52–34.38)	23.49 (12.76–32.27)	0.4
		14.8–71.55	2.71–43.99	
*p* (B-WRP vs. P-WRP)		0.201	0.041	
IL-8	B-WRP	18.72 (12.7–50.53)	12.52 (9.7–27.49)	0.263
		6.06–93.32	8.13–76.87	
	P-WRP	24.84 (12.19–44.68)	19.34 (15.54–47.45)	0.845
		8.49–67.8	2.71–180.04	
*p* (B-WRP vs. P-WRP)		0.149	0.209	
IL-12	B-WRP	6.41 (4.92–14.37)	5.77 (3.16–14.58)	0.54
		3.19–36.69	1.56–20.75	
	P-WRP	8.58 (4.82–15.32)	6.78 (3.67–12.53)	0.488
		1.2–43.62	2.32–27.8	
*p* (B-WRP vs. P-WRP)		0.57	0.937	
IL-17	B-WRP	2.01 (1.05–4.97)	2.23 (1.21–4.2)	0.863
		0.01–48.54	0.66–14.66	
	P-WRP	2.66 (1.49–3.24)	2.69 (2.18–2.74)	0.956
		0.31–17.99	1.7–2.76	
*p* (B-WRP vs. P-WRP)		0.575	0.465	
TNF-α	B-WRP	27.11 (21.99–37.61)	18.14 (12.08–25.49)	0.039 *
		9.97–117.58	9.97–103.68	
	P-WRP	22.24 (18.08–25.9)	10.1 (8.92–16.07)	0.002 *
		9.71–64.05	2.71–88.44	
p (B-WRP vs. P-WRP)		0.018 *	0.019 *	

Legend: B-WRP—before weight-reduction program; P-WRP—post-weight-reduction program; IL—interleukin; TNF-α—Tumor Necrosis Factor alpha; *—significant *p*-value.

## Data Availability

The database of aggregated statistics ready for analysis is stored in a secure, confidential, and password-protected repository on the server of the Medical University of Silesia. The data were anonymized. Completely non-identifiable records may be made available to interested persons/organizations upon request at jzalejskafiolka@sum.edu.pl.

## Abbreviations

The following abbreviations are used in this manuscript:

BMI	Body Mass Index
BMR	Basal metabolic rate
B-WRP	Before weight-reduction program
CPM	Total daily energy expenditure
DDE	Daily energy deficit
FFM	Fat-free mass
Glc	Fasting glucose
HbA1c	Glycated hemoglobin A_1c_
HDL-C	Cholesterol in high-density lipoproteins
HOMA-IR	Homeostatic Model Assessment of Insulin Resistance
CH	Total cholesterol
ICO	Index of central obesity
ICMJE	International Committee of Medical Journal Editors
IL	Interleukin
IL-1ra	Interleukin-1 receptor antagonist
IŻŻ	Polish National Food and Nutrition Institute
LDL-C	Cholesterol in low-density lipoproteins
MCP-1	Monocyte Chemoattractant Protein-1
MDA	Malondialdehyde
new-AIP	Modified Atherogenic Index of Plasma
OSI	Oxidative Stress Index
P-WRP	Post-weight-reduction program
PBF	Percentage of body fat
PWO	People with obesity
SMM	Skeletal-muscle mass
TAC	Total antioxidant capacity
TNF-α	Tumor Necrosis Factor alpha
TBW	Total body water
Th	T helper cell
TOS	Total oxidant status
VFA	Visceral fat area
WHO	World Health Organization
WHR	Waist-to-hip ratio
WRP	Weight-reduction program

## References

[1] World Health Organization Obesity and Overweight. https://www.who.int/news-room/fact-sheets/detail/obesity-and-overweight.

[2] (2025). GBD 2021 Adult BMI Collaborators. Global, regional, and national prevalence of adult overweight and obesity, 1990–2021, with forecasts to 2050: A forecasting study for the Global Burden of Disease Study 2021. Lancet.

[3] Weisberg S.P., McCann D., Desai M., Rosenbaum M., Leibel R.L., Ferrante A.W. (2003). Obesity is associated with macrophage accumulation in adipose tissue. J. Clin. Investig..

[4] Hotamisligil G.S. (2006). Inflammation and metabolic disorders. Nature.

[5] Ouchi N., Parker J.L., Lugus J.J., Walsh K. (2011). Adipokines in inflammation and metabolic disease. Nat. Rev. Immunol..

[6] Lumeng C.N., Saltiel A.R. (2011). Inflammatory links between obesity and metabolic disease. J. Clin. Investig..

[7] Gregor M.F., Hotamisligil G.S. (2011). Inflammatory mechanisms in obesity. Annu. Rev. Immunol..

[8] Crasan I.-M., Tanase M., Delia C.E., Gradisteanu-Pircalabioru G., Cimpean A., Ionica E. (2025). Metaflammation’s role in systemic dysfunction in obesity: A comprehensive review. Int. J. Mol. Sci..

[9] Wu H., Ballantyne C.M. (2020). Metabolic inflammation and insulin resistance in obesity. Circ. Res..

[10] Chehimi M., Vidal H., Eljaafari A. (2017). Pathogenic role of IL-17-producing immune cells in obesity and related inflammatory diseases. J. Clin. Med..

[11] Zúñiga L.A., Shen W.J., Joyce-Shaikh B., Pyatnova E.A., Richards A.G., Thom C., Andrade S.M., Cua D.J., Kraemer F.B., Butcher E.C. (2010). IL-17 regulates adipogenesis, glucose homeostasis, and obesity. J. Immunol..

[12] Gado M., Tsaousidou E., Bornstein S.R., Perakakis N. (2024). Sex-based differences in insulin resistance. J. Endocrinol..

[13] Vijayakumar S., Pantha S., Budha Magar S., Dhakal S. (2026). Sex differences in the impact of obesity on immunity. Immunol. Rev..

[14] Montefusco L., D’Addio F., Loretelli C., Ben Nasr M., Garziano M., Rossi A., Pastore I., Plebani L., Lunati M.E., Bolla A.M. (2021). Anti-inflammatory effects of diet and caloric restriction in metabolic syndrome. J. Endocrinol. Investig..

[15] Bulmer C., Avenell A. (2025). The effect of dietary weight-loss interventions on the inflammatory markers interleukin-6 and TNF-alpha in adults with obesity: A systematic review and meta-analysis of randomized controlled clinical trials. Obes. Rev..

[16] Muhammad H.F.L., Pahdarina D., Zahara N.P., Nugraheni F., Hanny T.A., Ermamilia A., Huriyati E. (2021). Diet or exercise: The role of diet and/or exercise on changes of pro-inflammatory markers during a weight loss program in adult women with overweight. Clin. Nutr. ESPEN.

[17] Zalejska-Fiolka J., Birková A., Wielkoszyński T., Hubková B., Szlachta B., Fiolka R., Błaszczyk U., Kuzan A., Gamian A., Mareková M. (2022). Loss of skeletal muscle mass and intracellular water as undesired outcomes of weight reduction in obese hyperglycemic women: A short-term longitudinal study. Int. J. Environ. Res. Public Health.

[18] Zalejska-Fiolka J., Birková A., Hubková B., Čižmárová B., Szlachta B., Fiolka R., Błaszczyk U., Wylęgała A., Kasperczyk S., Grzanka A. (2022). Successful correction of hyperglycemia is critical for weight loss and a decrease in cardiovascular risk in obese patients. J. Nutr. Biochem..

[19] Szlachta B., Birková A., Wielkoszyński T., Gospodarczyk A., Hubková B., Dydoń M., Zalejska-Fiolka J. (2023). Serum oxidative status in people with obesity: Relation to tissue losses, glucose levels, and weight reduction. Antioxidants.

[20] Szlachta B., Birková A., Čižmárová B., Głogowska-Gruszka A., Zalejska-Fiolka P., Dydoń M., Zalejska-Fiolka J. (2024). Erythrocyte oxidative status in people with obesity: Relation to tissue losses, glucose levels, and weight reduction. Antioxidants.

[21] Petralli G., Salvati A., Tricò D., Ricco G., Colombatto P., Brunetto M.R., Solini A. (2024). Simple proxies of insulin resistance identify obese metabolic dysfunction-associated fatty liver disease subjects with advanced liver disease. Diabetes Metab. Res. Rev..

[22] Isokuortti E., Zhou Y., Peltonen M., Bugianesi E., Clement K., Bonnefont-Rousselot D., Lacorte J.-M., Gastaldelli A., Schuppan D., Schattenberg J.M. (2017). Use of HOMA-IR to diagnose non-alcoholic fatty liver disease: A population-based and inter-laboratory study. Diabetologia.

[23] Zalejska-Fiolka J., Hubková B., Birková A., Veliká B., Puchalska B., Kasperczyk S., Błaszczyk U., Fiolka R., Bożek A., Maksym B. (2019). Prognostic value of the modified atherogenic index of plasma during body mass reduction in Polish obese/overweight people. Int. J. Environ. Res. Public Health.

[24] Erel O. (2005). A new automated colorimetric method for measuring total oxidant status. Clin. Biochem..

[25] Erel O. (2004). A novel automated direct measurement method for total antioxidant capacity using a new generation, more stable ABTS radical cation. Clin. Biochem..

[26] Carvalho J.B., de Andrade G.K.P., do Nascimento L.A., Golin N., Rodrigues A.L.C.C., Suiter E., Soprani M.V.O., Nadolskis A.S. (2023). Visceral fat area measured by electrical bioimpedance as an aggravating factor of COVID-19: A study on body composition. BMC Infect. Dis..

[27] (2015). Quality Management Systems—Requirements.

[28] (2016). Medical Devices—Quality Management Systems—Requirements for Regulatory Purposes.

[29] (2014). Medical Electrical Equipment—Part 1: General Requirements for Basic Safety and Essential Performance.

[30] (2015). Medical Electrical Equipment—Part 1-2: General Requirements for Basic Safety and Essential Performance—Collateral Standard: Electromagnetic Disturbances—Requirements and Tests.

[31] CE MDD (Directive 93/42/EEC) (1993). Council Directive 93/42/EEC of 14 June 1993 Concerning Medical Devices.

[32] de Baat A., Trinh B., Ellingsgaard H., Donath M.Y. (2023). Physiological role of cytokines in the regulation of mammalian metabolism. Trends Immunol..

[33] Petersen A.M.W., Pedersen B.K. (2005). The anti-inflammatory effect of exercise. J. Appl. Physiol..

[34] Ballak D.B., Stienstra R., Tack C.J., Dinarello C.A., van Diepen J.A. (2015). IL-1 family members in the pathogenesis and treatment of metabolic disease: Focus on adipose tissue inflammation and insulin resistance. Cytokine.

[35] Donath M.Y. (2013). Targeting inflammation in the treatment of type 2 diabetes. Diabetes Obes. Metab..

